# Improving Health Promotion Using Quality Improvement Techniques in Australian Indigenous Primary Health Care

**DOI:** 10.3389/fpubh.2016.00053

**Published:** 2016-03-30

**Authors:** Nikki Percival, Lynette O’Donoghue, Vivian Lin, Komla Tsey, Ross Stewart Bailie

**Affiliations:** ^1^Centre for Primary Health Care Systems, Menzies School of Health Research, Brisbane, QLD, Australia; ^2^Department of Public Health, School of Psychology and Public Health, LaTrobe University, Melbourne, VIC, Australia; ^3^The Cairns Institute, James Cook University, Cairns, QLD, Australia

**Keywords:** health promotion, quality improvement, Indigenous, primary health care, evidence-based program, feasibility, participatory action research

## Abstract

Although some areas of clinical health care are becoming adept at implementing continuous quality improvement (CQI) projects, there has been limited experimentation of CQI in health promotion. In this study, we examined the impact of a CQI intervention on health promotion in four Australian Indigenous primary health care centers. Our study objectives were to (a) describe the scope and quality of health promotion activities, (b) describe the status of health center system support for health promotion activities, and (c) introduce a CQI intervention and examine the impact on health promotion activities and health centers systems over 2 years. Baseline assessments showed suboptimal health center systems support for health promotion and significant evidence-practice gaps. After two annual CQI cycles, there were improvements in staff understanding of health promotion and systems for planning and documenting health promotion activities had been introduced. Actions to improve best practice health promotion, such as community engagement and intersectoral partnerships, were inhibited by the way health center systems were organized, predominately to support clinical and curative services. These findings suggest that CQI can improve the delivery of evidence-based health promotion by engaging front line health practitioners in decision-making processes about the design/redesign of health center systems to support the delivery of best practice health promotion. However, further and sustained improvements in health promotion will require broader engagement of management, senior staff, and members of the local community to address organizational and policy level barriers.

## Introduction

The disparities between the health status of Aboriginal and Torres Strait Islander people (Australia’s Indigenous populations) and that of other Australians is unacceptable. Indigenous Australians have a life expectancy 10.6 and 9.5 years lower than that of non-Indigenous males and females, respectively, infant mortality is three times higher, and death rates are 1.6 times that of other Australians (1). Although there have been improvements in some social and health indicators (2), chronic diseases, such as cardiovascular disease, diabetes, and renal disease, remain significant contributors to premature and excess mortality and morbidity among Indigenous Australians.

Although the root causes of poor and inequitable health are related more to social, cultural, and environmental factors, the health sector is a vital determinant of health and plays a key role in promoting equity (3) and supporting action to address social determinants of health (4, 5). International experience has shown the positive effect of health systems based on equity, disease prevention, and health promotion in narrowing health inequities (6) and more specifically, in reducing Indigenous health inequities. For example, access to an integrated and comprehensive primary health care (PHC) system, with a strong primary and preventive focus, has been critical in delivering better health for Native Americans and the Maori people of New Zealand (7).

Comprehensive PHC services in Australia are best typified by the Aboriginal community controlled health services (ACCHS). These health services are designed to deliver holistic, comprehensive, and culturally appropriate health care for Indigenous Australians. The National Aboriginal Community Controlled Health Organisation (NACCHO) describes PHC as including not only the provision of medical care but also the provision of services, such as counseling, preventive medicine, health education and promotion, rehabilitative services, antenatal and postnatal care, and maternal and child care programs (8). Although health promotion is recognized as a core function, there has been little published research that has considered the health promotion work of these PHC centers (9).

In the Australian Indigenous PHC context, there is growing appreciation of both the need for and benefits of using continuous quality improvement (CQI) techniques to improve the delivery of a range of PHC services through an emphasis on organizing and strengthening fragmented health systems (10). Sollecito and Johansen (11) define CQI as “*a structured organisational process for involving staff in planning and executing a continuous flow of improvements to provide quality that meets or exceeds the expectations of customers*.” It involves designing and redesigning systems to meet customers’ needs by testing and implementing ideas from evidence-based strategies, frontline staff, and customers. Although there has been substantial research on CQI in clinical health care in Australian Indigenous communities, the study of quality improvement in health promotion has been limited.

We conducted a 3-year study exploring the potential of CQI for improving health promotion in collaboration with Indigenous PHC centers in Australia’s Northern Territory (NT). Combining the Ottawa Charter for Health Promotion’s definition of “health promotion” and the NACCHO’s definition of “health,” we define health promotion as “the process of enabling Indigenous people to increase control over, and to improve, not just the physical wellbeing of the individual, but the social, emotional, and cultural wellbeing of the whole community in which each individual is able to achieve their full potential as a human being, thereby bring about the total wellbeing of their community” (8, 12). The term “health education” can sometimes be used synonymously with health promotion. To emphasize the distinction, and for the purpose of this study, we consider health education an important and common strategy in health promotion; defined as “the provision of education to individuals (through discrete planned sessions) or groups, with the aim of improving knowledge, attitudes, self-efficacy and individual capacity to change” (13) (p. 20). The study involved developing and implementing health promotion CQI tools and processes with the aim of assisting health center staff to design/redesign their health center systems and strengthening the development and delivery of local health promotion activities. Using the health promotion CQI tools and processes, the purposes of this study were to (a) describe the scope and quality of health promotion activities, (b) describe the status of health center system support for health promotion activities, and (c) examine the impact of a CQI intervention on health promotion activities and health centers systems after two annual cycles.

## Materials and Methods

### Study Setting and Processes

Primary health care centers participating in this study are located in regional and remote Aboriginal communities in Australia’s NT. The NT is in the central northern region of Australia and spans 1.3 million square kilometers, making it the third largest Australian federal division. However, it is sparsely populated, with an estimated population of 243,800, making it the least populous of Australia’s eight states and territories (14).

The NT has the highest proportion of Indigenous Australian residents estimated at 68,850 or 29.8% of the total NT population compared to 3% of the total Australian population (15). About 90% of the NT’s Indigenous populations live in discrete, remote communities (15). In remote communities, access to health care is predominately through Indigenous-specific PHC services, including Aboriginal community-controlled or state government PHC centers. There is seldom more than one PHC provider. This differs from most other Australians who can access PHC services through a fee-for-service sector based on general medical practice and a state government-funded and managed sector, which differs from state to state in its forms and functions.

Two separate but complementary governance structures were developed specifically for this study. A project management committee was established comprising the lead study investigators, members of the research team, and senior policy officers and managers from the Northern Territory Health Department (NTDoH). The project management committee was responsible for guiding the research processes and maintaining academic rigor. An Aboriginal and Torres Strait Islander Advisory Committee acted as a reference group for the study, assisting in the development of the data collection tools and facilitating health center and community engagement.

All research procedures related to this study were approved by the Human Research Ethics Committee of the NT Department of Health and Families and Menzies School of Health Research, and by Menzies Indigenous health research subcommittee. Formal participation agreements setting out the roles and responsibilities of the research team and those of health centers and staff (including issues relating to data collection and storage, confidentiality, intellectual property, and research dissemination) were negotiated and signed by health center management and, where appropriate, by health boards. Prior to each site visit, clearance was provided by the relevant Aboriginal community council and local health center.

### Development and Implementation of the CQI Intervention

**Figure 1:**
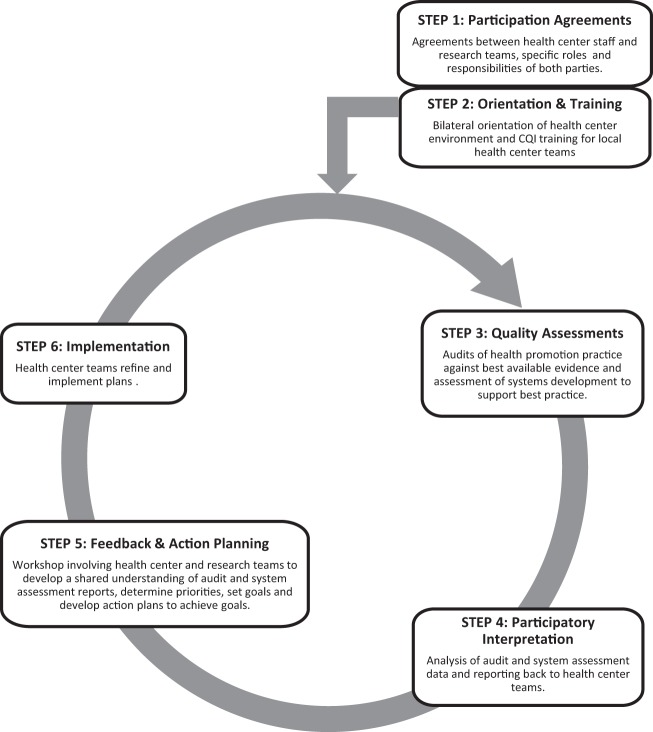
**The health promotion continuous quality improvement model [adapted from Bailie et al. (10)]**.

The health promotion CQI intervention was delivered through an action research design, whereby health center staff, the research team, and other key stakeholders (e.g., Indigenous and non-Indigenous health promotion practitioners and policy officers) were involved in the development and refinement of existing CQI tools that have been extensively used for the improvement of clinical care in Indigenous PHC settings (16). Experience gained and the results of regular data feedback were used to continually revise and improve the tools and processes for health promotion. By drawing on principles of participatory action learning (17) and guidelines for the ethical conduct in Aboriginal and Torres Strait Islander health research (18), our approach aimed to maximize engagement of stakeholders at multiple levels, from local health center management and PHC staff to policy decision makers. The CQI intervention is summarized in Figure 1. It comprised annual cycles of health center systems’ assessments and audits of local health promotion activities; data analyses and interpretation; feedback and local interpretation of results with participating health center staff; goal setting by health center staff to achieve system changes; action planning; and strategy implementation.

### Data Sources and Methods

The research team and local health center staff used the health promotion audit tool (available from: http://www.one21seventy.org.au/cqi-information/hp-cqi-tools) and audit protocol to review health center records of health promotion activities (for example, project plans, staff reports and presentations, and minutes of meetings) that had been implemented in the preceding 12 months for documentation of key aspects of health promotion planning, implementation, and evaluation. Audits were conducted at baseline and annually for the next 2 years. Details on the audit method have been described previously (19).

The audit and best practice in chronic disease (ABCD) systems assessment tool (SAT) (20) was adapted and used to guide the assessment of health center system support for health promotion. It comprised an interactive process whereby the research team engaged health center staff [health center managers, nurses, Aboriginal health workers (AHWs), and doctors when available] in discussion and reflection on the strengths and weaknesses of how their health center is organized and functions. They were encouraged to consider the systems currently in place to support planning and delivery of health promotion and to comment on the successes and difficulties.

At the end of each data collection cycle, the research team drew on the findings to facilitate a structured reflection with the health center team about potential actions for system improvement. This feedback process usually occurred within 3 months of data collection and within a timeframe that enabled a response to influence the next cycle. This participatory feedback process was a key component of the action research methodology.

### Analysis

The types of health promotion activities were categorized on a continuum from health education and skill development activities; health information and social marketing activities; community development activities designed to strengthen capacity of communities to address local health issues; and activities designed to improve healthy environments, settings, and to change socio-environmental causes of disease (21). Improvements in health promotion activities over the study period (as represented by the number and percentage of activities that provided documentary evidence of key aspects of best practice) were assessed by comparing the Year 1 data (collected after one annual cycle) and Year 2 data (collected after two annual cycles) with baseline data. Matrix displays were used as a way of organizing and visualizing qualitative data collected through the SAT in a systematic way and comparing improvements over time and across participating health centers (22). The research team met to identify and discuss patterns and themes in the descriptions of systems and classified them as a strength (system working well) or weakness (system not working so well) for supporting health promotion for each component at participating PHC centers. These emerging findings were presented to health center staff during annual feedback workshops and to investigators and stakeholders at quarterly project meetings, where interpretations were discussed to check their validity. All health centers were provided final reports describing the results of the health promotion quality assessments and changes over time that are presented in this paper.

## Results

### Characteristics of Participating Health Centers

From January 2008 to December 2010, four Indigenous PHC centers were engaged in the study. Table 1 shows their diversity with regard to governance arrangements, geography, and population size. Three health centers are governed by a board of elected Indigenous community members (community controlled) and one health center is managed and operated by the NTDoH (government service). Two health centers deliver PHC services to populations of more than 1,000 people (but less than 5,000), one health center delivers PHC services to more than 5,000 people, and one delivers PHC services to a population of less than 500 people.

**Table 1:** **Characteristics of participating Indigenous primary health care centers**.

Health center	Health center governance	Population size^a^	Remoteness^b^
A	Government	1,486	(i) Part year by road
			(ii) 301–600 km by road
B	Community controlled	2,156	(i) All year by air or sea
			(ii) By air
C	Community controlled	9,022	(i) All year by road
			(ii) <20 km by road
D	Regional health board	319	(i) All year by road
			(ii) 20–100 km by road

*^a^Total population: estimates only (23)*.

*^b^Remoteness (24) (i) access to community: all year by road; part year by road; and all year by air or sea (islands) and (ii) distance to urban center: <20 km by road; 20–100 km by road; 101–300 km by road; 301–600 km by road; and by air (islands)*.

Each health center employs multidisciplinary teams of 5 to more than 50 staff including nurses, allied health workers, doctors, and AHWs. (AHWs are often recruited from their remote communities enabling local participation in the direction and delivery of health services. Although AHWs exist in all Australian states and territories, the title AHW covers many roles. In the NT, the title AHW is regulated in recognition of the specific scope of work practices, particularly clinical work, ensuring that work is carried out without risk to public safety.) With the exception of one health service, staff employed in these health centers were not specialist health promotion practitioners. One health center had appointed a health promotion coordinator approximately 6 months prior to the commencement of this study.

Data about the scope and quality of health promotion activities were gathered from a total of 51 health promotion activities across the four PHC centers. The number of activities audited in each of the 3 years was 8 in 2008 (baseline), 24 in 2009 (Year 1), and 19 in 2010 (Year 2). A total of 11 systems assessments were conducted over the course of the study. Each health center had an annual systems assessment, however in Year 2, the final round of data collection, one health center declined to participate in the SAT due to changes in management and staffing at that time. We facilitated annual feedback workshops with each health center. Some health centers were more proactive than others in documenting their action plans for improvement (see Figure 1, step 5).

### Baseline Results

#### Scope and Quality of Health Promotion Activities

At baseline, the type of health promotion activity was dominated by health education (three activities) and information sessions (three activities), with less emphasis on community development activities (one activity), or activities designed to change socio-environmental causes of disease (one activity). All health centers delivered activities to raise community awareness about the risks of smoking and other common chronic disease risk factors (nutrition, alcohol, and physical activity). Other targeted areas included diabetes, chronic obstructive pulmonary disease, and hypertension.

Written descriptions of activities in health center records were limited (two out of eight activities) and inadequate for collecting information related to planning, implementation, and evaluation of health promotion activities.

#### Health Center System Design and Support for Health Promotion

Baseline systems assessments showed that health promotion was organized in a variety of ways in PHC centers. Health promotion was considered a discrete area of program service delivery, as a part of all service delivery, or the responsibility of individual members of staff. Some health center staff described visiting and external services as part of their “health promotion delivery team.” Health promotion was articulated as a core function of PHC service delivery in some but not all health center strategic plans and/or mission statements.

Staff reported significant perceived weaknesses in health center system support for health promotion. Across health centers, the majority of staff held the view that staffing levels to support the design and implementation of health promotion activities was inadequate and that pressure from “the clinic” mitigated their ability to undertake health promotion. Staff reported their roles and responsibilities (including reporting and communication) for health promotion were not always clearly defined or perceived as an implicit part of their role.

Across and within each health center, staff had different perspectives of “health promotion” and how it is done. Health center staff used the phrases “health education” and “health promotion” interchangeably, as if they were one and the same. Health center staff commented that health promotion language or “jargon” was a major obstacle in understanding and applying principles and concepts in practice.

Systems (or alternative processes) that could be used to collate, report, and monitor local health promotion activities were limited. Existing service planning and monitoring systems did not reflect, or are only partially supportive of, health promotion. For example, patient information systems (either paper based or electronic) designed for delivery of clinical services and health care to individuals had limited capacity to support the design, implementation, and evaluation of health promotion activities.

### Actions for Improving Health Promotion

Based on findings from the annual cycles, health center staff identified and implemented a range of actions to improve health center system support for health promotion. Summarized in Table 2, these actions address four broad system components.

**Table 2:** **Examples of actions implemented by health center teams to improve health center systems support for health promotion**.

**Delivery system design**
Included “health promotion” as an agenda item at weekly staff meetings
Arranged health promotion portfolios for all staff
Identified two Aboriginal health workers to form a health promotion team
Senior Aboriginal health worker designated as “broker” between the local community and health service
Appointed a staff member to coordinate training and professional development in health promotion for staff
**Information systems and decision support**
Created arch lever folders for storing documents and records for health promotion
Developed standardized planning templates and trialed quality improvement program planning system (QIPPS)
Used the Health Promotion Audit Tool as a “check list” for documenting practice
Community board representatives attend feedback sessions
Purchasing of best practice guidelines (e.g., *The Public Health Bush Book*)
**Organizational environment**
Workshops/trainings in health promotion made available to staff
Results of health promotion audit presented to health board
Management quarantined time for staff to participate in health promotion CQI processes
Health board chair invited and participated in the CQI feedback workshop
Involvement of external practitioners in health promotion CQI processes
**Adaptability and integration of health system components**
Create referral pathway in existing clinical information systems to capture group health education sessions
Sharing “good practice” health promotion plans across health center teams
Health promotion officers from NT Department of Health support health service staff to access and use the quality improvement program planning system (QIPPS) to plan health promotion activities
Using clinical service data to develop health promotion project (e.g., storyboard for HbA1c)

#### Delivery System Design

To improve the way health promotion was delivered and by whom, staff initiated strategies to create greater clarity of individual roles and responsibilities in health promotion and improve communication about health promotion within the health center. For example, at health center D, health promotion was added as an agenda item at weekly staff meetings to improve communication about staff involvement in local activities. Health center A incorporated health promotion as part of each program area portfolio and health center B identified two AHWs to form a health promotion team. These actions were designed to improve staff understanding of their roles and responsibilities in health promotion at their respective health centers.

#### Information Systems and Decision Support

“Information systems and decision support” refers to health center structures and processes that support planning, implementation, and monitoring of health promotion activities. This includes access to evidence-based tools and guidelines and systems for recording and monitoring health promotion activity. At baseline, systems or processes to record and monitor health promotion were lacking. Over the course of the study, health center teams introduced a range of systems to standardize documentation and recording of health promotion activities. For example, health centers C and D trialed the quality improvement program planning system (QIPPS) – an electronic program planning information system (see http://www.qipps.infoxchange.net.au). Health centers A and B introduced paper-based planning templates (using items in the audit tool) to document practice. Additionally, at health center A, staff trialed ways to document health promotion in their existing clinical information systems.

#### Organizational Environment

Actions to improve the broader health center environment for health promotion were focused on raising awareness and engaging senior staff and health board members in health promotion. Baseline analysis revealed that health center staff were aware of the importance of community involvement in health promotion activities; however, the mechanisms for supporting this action, such as community boards and advisory committees, did not exist or were not used for the purpose of strengthening health promotion at the health center. For example, three out of the four participating health centers are governed by a health board made up of community representatives. Results from systems analyses highlighted that some staff perceived the role of the board as decision makers regarding services “at the clinic” and had not considered their involvement or understood their role for supporting health promotion. Over the study period, staff invited board members to participate in data collection and feedback workshops. At health center D, a staff representative presented at a board meeting and discussed audit results with community representatives.

#### Adaptability and Integration of System Components

To ameliorate fragmented health center systems, staff identified a number of strategies to integrate and link different system components. For example, health center staff utilized data from other quality improvement initiatives to inform the development of local health promotion activities. At health center B, a number of patients with type 2 diabetes were identified as having elevated levels of glycated hemoglobin (HbA1c). Based on the traditional ways of storytelling [see “Chronic Disease Storyboard” in Laycock et al. (25)], AHWs developed health education sessions to raise community awareness and understanding of sugar consumption and its impact on the development and management of diabetes.

### Changes in Scope and Quality of Health Promotion Activities Overtime

Table 3 presents a summary of the aggregated audit data across the four PHC centers. Following the introduction of the CQI intervention, we observed improvements in aspects of planning, implementation, and evaluation of health promotion activities. However, within and across health centers, the type of health promotion activities and targeted health issues remained largely unchanged over the study period.

**Table 3:** **Results from audits of health promotion activities at baseline, Year 1, and Year 2 across four participating health centers [figures are number and percentage (%) of activities]**.

Documentation of health promotion activities	Baseline (*n* = 8)	Year 1 (*n* = 24)	Year 2 (*n* = 19)
**Planning**			
Number and percentage of activities that had documented health promotion plans	2/8 (25%)	19/24 (79%)	17/19 (89%)
**Targeting**			
Number and percentage of activities that recorded the target group	1/8 (13%)	19/24 (79%)	15/19 (79%)
Number and percentage of activities that recorded the delivery setting	1/8 (13%)	18/24 (75%)	12/19 (63%)
Number and percentage of activities that recorded attempts to address chronic disease-related behaviors	2/8 (25%)	21/24 (88%)	10/19 (53%)
**Community participation**			
Number and percentage of activities that recorded community participation	1/8 (13%)	9/24 (37%)	7/19 (37%)
**Partnerships**			
Number and percentage of activities that recorded partnerships with outside agencies and organizations	1/8 (13%)	13/24 (54%)	12/19 (63%)
**Evaluation**			
Number and percentage of activities that had documented an evaluation	3/8 (38%)	11/24 (46%)	11/19 (58%)

When compared with baseline, health centers had improved documentation plans for their health promotion activity from 2/8 (25%) at baseline to 17/19 (89%) at Year 2. Improvements in documenting aspects of planning and evaluation were also found. At Year 2, 14/19 (74%) health promotion activities had recorded an activity goal; 15/19 (79%) had recorded the activity target group; 16/19 (84%) recorded strategies for implementing the activity, and for 11/19 (58%) health promotion activities there was evidence of activity evaluation.

Prior to the CQI intervention, recorded participation of community people in health promotion activities was low (1/8; 13%). At Year 1, improvements in community involvement was noted with a record of community participation in 9/24 (37%) activities. There was no further change in recorded community participation in health promotion activities at Year 2 (7/19; 37%).

Our findings also highlight the extent to which health center teams worked with other organizations. At baseline, of the eight health promotion activities, only one recorded involvement of other organizations (13%) in the planning, implementation, and evaluation of health promotion. Of the 24 activities in Year 1 and 19 activities in Year 2, partners were involved in just over half (54%) of the activities in Year 1 and slightly more activities (63%) in Year 2. Partnerships were with organizations from the health sector (for example, state government, national government, and non-government health services and health-related aid organizations).

## Discussion and Conclusion

Our study showed that the introduction of structured and facilitated quality improvement cycles can improve health center systems and quality of health promotion activities in Indigenous PHC centers. At baseline, we found that PHC centers undertake health promotion activities, but what health promotion has done was often not recorded, or when documented, the information was scarce and not comprehensive. We also found that the activities were largely dominated by lifestyle advice and education approaches, and responding to growing levels of chronic disease was the focus of their efforts. Community participation in the planning, implementation, and evaluation of activities was also limited and showed little improvement over time.

The CQI intervention appears to have contributed to an increase in the number and quality of health promotion activities by improving health center staff health promotion capacity and system development and functioning. For example, through PHC, staff are better able to articulate their health promotion work and, subsequently, identify and document health promotion activities; improve systems for recording health promotion activities, thereby enhancing availability of data; and, subsequently, improve workforce capacity to deliver health promotion activities over the study period. The health promotion audit tool is based on health promotion planning and evaluation frameworks and, as such, appears to have assisted health centers to reflect upon the extent to which they incorporate and, subsequently, document these concepts and principles into their health promotion activities.

Limited attention to areas of system development that support aspects related to the “process” of health promotion may provide some explanation for limitations in delivery and recordings of other areas of health promotion activity quality. For example, records of community participation in health promotion activities improved from baseline to Year 1, but no further improvements were achieved in Year 2. Although it may be desirable to improve the ecological approach of health promotion activities (26, 27), the health promotion skills and expertise and the time required to effectively and meaningfully engage community people and partners in this process is likely to be well beyond currently available capacity in the PHC centers. Thus, the importance of a coordinated and partnered approach to health promotion in these communities becomes even more critical if health promotion is to be effective, and action on the social determinants of health is to be realized. A potential approach for coordinating health promotion activities is through the inclusion of relevant stakeholders, such as representatives of the governing health board, other organizations and agencies in the community, and visiting services, throughout the CQI intervention. This would help to avoid duplication of effort and improve local planning processes for health promotion.

Another key finding of this study is that participating PHC health centers focused actions for systems improvement on transactional system change; that is, the day-to-day operations of the organization (28). Improvements were most seen in areas of delivery system design and information and decision support, such as through the introduction of standard templates for recording health promotion activities, purchase of resources to guide practice, and creation of team portfolios for health promotion. Although these are necessary and important system improvements for supporting health promotion activities, broader transformational changes that are more closely linked with leadership, vision, organizational culture, and external environments are necessary if health promotion is to be a core component of PHC service delivery (28). Organizational change of this nature is possible as has been demonstrated for diabetes care (20) and for health promotion (29, 30). For these health centers to become more health promoting, actions to improve systems related to the organizational environment are an important area of influence and for future consideration.

Even though Australian Indigenous PHC centers have been described as exemplary models of comprehensive PHC (31) and that health promotion is a recognized core function of NT PHC centers (32), our findings, together with other studies of health promotion in PHC settings (9, 33, 34), suggest that health centers struggle to implement health promotion as a core component of their service delivery. Notwithstanding health center agreements to participate in this study, and with the exception of a few staff directly involved in health promotion activities, overwhelmingly, health center staff felt overloaded with issues of patient care and delivery of clinical services. This was evident from SAT data but also expressed by the lack of attendance of some health center managers and senior staff in the CQI process. The disparate availability and/or allocation of resources, including health promotion positions or skilled staff, available time, and funding, further suggests that health promotion is not an integral component of PHC service delivery. However, what is clear from our study is that participation in the CQI process gives health center teams’ dedicated time to discuss and reflect on health promotion in their health center. With a better understanding of what constitutes good practice and knowledge of health systems that support optimal practice, health center teams can not only build new systems but also identify the potential of existing structures. Even under challenging circumstances, health center teams can take small, incremental steps toward establishing partnerships with local organizations and engage community in aspects of health promotion planning, implementation, and evaluation. This is particularly important in resource constrained environments.

We acknowledge that the use of health center records for assessment purposes may have limited the number of health promotion activities included in the study and recognize that the health center teams may be involved in other activities not captured here. However, we have used a variety of strategies to strengthen our findings, including the use of multiple methods and data sources, and seeking validation of our analysis with health center teams and project stakeholders throughout the study.

Availability and quality of health promotion records created challenges for conducting audits of health promotion and have flow on effects for health promotion practice, including the ability to monitor and to evaluate the impact of health promotion activities. The lack of documentation of health promotion activities (success or otherwise) further perpetuates the lack of evidence of effective health promotion, duplication of effort and repetition of activities with little to no effect, and an inability to “scale up” effective interventions.

The CQI intervention appears to be a useful strategy for identifying and subsequently improving several key areas of health promotion by engaging staff in the design and redesign of health center systems. We recognize the implementation of the CQI cycle is complex and requires investment of resources, both for facilitation and for the provision of relief staff time to allow all members of the health center team to fully participate in the quality improvement process. Previous research investigating health service involvement in CQI indicates that the commitment from health center managers, senior clinicians, and other leadership positions at the regional level is critical for creating an environment where staff can participate and actively engage in the CQI process (35). This level of commitment will also be crucial for expanding the health promotion capacity of the PHC workforce and for sustaining CQI interventions in health promotion.

Since funding for the original research project ended, we have embraced a range of innovative research translation activities to ensure the uptake of research findings in policy and practice. The health promotion audit and system assessment tools have been refined into web-based tools, resources, and a training package for use through the National Centre for Quality Improvement in Indigenous Primary Health Care (www.One21seventy.org.au). This research translation process enables health services, nationwide access to the quality improvement tools, training, and support to improve Indigenous health promotion. Recommendations from the study have informed the development of a NT implementation plan for quality improvement in Indigenous health promotion. We continue to collaborate with NTDoH in supporting the widespread uptake and implementation of the quality improvement tools and processes.

## Author Contributions

NP led drafting of the manuscript and development of the CQI tools and worked in data collection and analysis. LO facilitated engagement of health centers, played a key role in developing the CQI tools, worked in data collection and analysis, and ensured cultural appropriateness. RB, KT, and VL oversaw CQI tool development, data collection and analysis. RB played a lead role in the study design. All authors revised and approved the final manuscript.

## Conflict of Interest Statement

The authors declare that the research was conducted in the absence of any commercial or financial relationships that could be construed as a potential conflict of interest.
